# Degradation of biodegradable plastic mulch films in soil environment by phylloplane fungi isolated from gramineous plants

**DOI:** 10.1186/2191-0855-2-40

**Published:** 2012-08-02

**Authors:** Motoo Koitabashi, Masako T Noguchi, Yuka Sameshima-Yamashita, Syuntaro Hiradate, Ken Suzuki, Shigenobu Yoshida, Takashi Watanabe, Yukiko Shinozaki, Seiya Tsushima, Hiroko K Kitamoto

**Affiliations:** 1 National Institute for Agro-Environmental Sciences, 3-1-3 Kannondai, Tsukuba, Ibaraki, 305-8604, Japan; 2 Present address: National Agriculture and Food Research Organization, 3-1-1 Kannondai, Tsukuba, Ibaraki, 305-8517, Japan

**Keywords:** Biodegradable plastic, Leaf surface, Phylloplane fungi, Mulch film, PCR-DGGE

## Abstract

To improve the biodegradation of biodegradable plastic (BP) mulch films, 1227 fungal strains were isolated from plant surface (phylloplane) and evaluated for BP-degrading ability. Among them, B47-9 a strain isolated from the leaf surface of barley showed the strongest ability to degrade poly-(butylene succinate-*co*-butylene adipate) (PBSA) and poly-(butylene succinate) (PBS) films. The strain grew on the surface of soil-mounted BP films, produced breaks along the direction of hyphal growth indicated that it secreted a BP-degrading enzyme, and has directly contributing to accelerating the degradation of film. Treatment with the culture filtrate decomposed 91.2 wt%, 23.7 wt%, and 14.6 wt% of PBSA, PBS, and commercially available BP polymer blended mulch film, respectively, on unsterlized soil within 6 days. The PCR-DGGE analysis of the transition of soil microbial community during film degradation revealed that the process was accompanied with drastic changes in the population of soil fungi and *Acantamoeba* spp., as well as the growth of inoculated strain B47-9. It has a potential for application in the development of an effective method for accelerating degradation of used plastics under actual field conditions.

## Introduction

Agricultural mulch films, which are used for covering cultivated fields to maintain a stable soil temperature and to prevent weeds from growing, are the most common and highly consumed plastic products on agricultural farm lands. The widespread use of mulch films however, has led to an increase in environmental wastes particularly because the removal and disposal of used mulch films is highly cumbersome and consumes a lot of energy (Kyrikou and Briassoulis [2007]).

To address these environmental issues, biodegradable plastics (BPs) have been developed (Gross and Kalra [2002]). Unlike typical plastics, BPs can be degraded by microorganisms in the natural environment. To date, many aliphatic polyesters which can be degraded in compost and moist soils, such as poly-(butylene succinate) (PBS) and poly-(butylene succinate-*co*-butylene adipate) (PBSA) have been developed as BPs with excellent formability like polyethylene (Xu and Guo [2010]). Commercially available BP mulch films have PBS as the main component and are blended with various BPs to control their strength and degradability speed adequate for ‘self-destructs’ after their useful lives have ended.

The degradation of these materials, however, is gradual and cannot be easily controlled (Kyrikou and Briassoulis [2007]). Degradation speed of BP mulch films in agricultural fields is sometimes very slow, as it is largely affected by the environmental conditions. The desirable practical BP is a programmed degradable plastic that has adequate performance properties during its planned useful life time, and is 100% post-use biodegradable. The existence of such structured plastics has not been confirmed yet (Gross and Kalra [2002]; Kyrikou and Briassoulis [2007]). If some efficient BP-degrading microorganisms were available, farmers can use them or their enzyme to degrade the used BP mulch films immediately after use, thus enabling them to manage efficiently the schedule of their next planting. Also, they will be able to use stronger plastic products that are not easily broken during use, and could degrade them by enzyme treatment whenever necessary (Kitamoto et al. [2011]).

Only a few studies, however, have been published so far on the use of such microorganisms to enhance the degradation of used BP mulch films in soil environment (Kasuya et al. [2009]; Abe et al. [2010]). Kasuya *et al*. (Kasuya et al. [2009]) isolated a fungal strain NKM1712 from soil, and observed weight loss of poly butylene adipare-*co*-butylene terephtalate (PBAT) film with inoculation of this strain in soil. Scanning electron microscopy (SEM) observation of degraded film on soil and comparative studies of the BP film-degrading ability of isolated microorganisms demonstrated the contribution of fungi to microbial degradation of BP films (Sang et al. [2002]; Tan et al. [2008]; Kasuya et al. [2009]). Recent investigation of microbial communities in BP-degrading compost by using direct DNA extraction from compost followed by cloning and sequencing showed that Ascomycota comprised the most dominant group of microorganisms during the biodegradation of BP films (Sangwan and Wu [2008]; Sangwan et al. [2009]). These results suggested the important roles of fungi in BP degradation. Although BP mulch films are degraded in soil, it has been very difficult to isolate the effective BP film-degrading microorganisms from it. If rich sources of target microorganisms were found, isolation of suitable BP-degrading strains for actual use in the field would become easy. With these considerations, we attempted to isolate such effective BP-degrading microorganisms from the natural environments and use them to develop a method for accelerating BP film degradation in natural environment.

Both BPs (Xu and Guo [2010]) and the cuticular layer of phylloplane (Heredia [2003]) are reported to be made of polyesters of fatty acids. This fact has led us to speculate that phylloplane microorganisms may effectively degrade BPs. Following this idea, we recently discovered that many phylloplane yeasts on rice and vegetables have strong abilities to degrade PBS and PBSA mulch film (Kitamoto et al. [2011]). Since phylloplane is a typical habitat for various fungi (Park [1982]), and increments in the populations of filamentous fungi were observed during the PB degradation in the soil (Sang et al. [2002]), we have speculated that effective BP-degraders may also be present among phylloplane filamentous fungi.

Using the recently developed extraction of DNA method from agricultural soil (Hoshino and Matsumoto [2004]) and the newly selected primer pair sequences suitable for amplification of 18 S rDNA fragments from the fungal genome in Japanese soil (Hoshino and Morimoto [2008]), have made it possible to analyze the diversities of fungal communities in Japanese soil by polymerase chain reaction-denaturing gradient gel electrophoresis (PCR-DGGE) (Anderson and Cairney [2004]). With these newly established methods, we can observe the microbial transition in soil during BP degradation.

The aim of the present study was to isolate various phylloplane filamentous fungi, screen them for BP film-degrading ability on agarose medium, and examine how the selected strain contribute to the degradation process of BP film on the soil using SEM and PCR-DGGE.

## Materials and methods

### Substrates and chemicals

To select the BP-degrading strains from the phylloplane fungal isolates, we used emulsified PBSA (Bionolle EM-301, average molecular weight, 12 to 15 × 10^4^). To evaluate their solid polymer–degrading activity, we used PBSA film (Bionolle 3001 G) and PBS film (Bionolle 1001G), both of which have an average molecular weight of 20 to 25 × 10^4^ and a thickness of 20 μm. These materials available under the product name Bionolle were obtained from Showa Denko K. K. (Tokyo, Japan).

To observe mulch film degradation on a soil surface, a commercially available biodegradable mulch film (20 μm thickness) was used. This mulch film is composed of succinate, adipate, and terephthalate at a molar ratio of 11.6:2.1:1.0, and made of PBS, PBSA, and PBAT at a butanediol monomer-based molar ratio of 49:37:14 (weight-based ratio of 47:37:17), which was determined by the liquid-state nuclear magnetic resonance (NMR) technique, as described below.

The mulch film and standard pellet samples of PBS (Bionolle 1020), PBSA (Bionolle 3020), and PBAT (Ecoflex F blend C1200, BASF AG, Germany) were dissolved in chloroform-*d* (CDCl_3_), and ^1^ H and ^13^ C NMR signals were determined at 600 and 151 MHz by using a NMR spectrometer (Alpha 600, JEOL, Tokyo, Japan). Chemical shifts were quoted with respect to tetramethylsilane but were determined by referring to the residual ^1^ H and ^13^ C signals of the solvent (chloroform). The ^13^ C NMR spectrum was used for assignment of the signals, and the ^1^ H NMR spectrum was used for determining the molar ratio of the monomers and the ratio of PBS, PBSA, and PBAT in the mulch film.

All chemicals used were analytical grade and obtained from Sigma-Aldrich (St. Louis, MO, USA) or Wako Chemicals, (Osaka, Japan).

### Strains and media

The strain B47-9 isolated in this study is deposited in the National Institute of Technology and Evaluation (NITE, Japan), NITE Patent Microorganisms Depositary (accession number: NITE P-573).

The media used for isolation of phylloplane fungi from leaf surfaces of gramineous plants, preservation of the isolated BP-degrading strains, and evaluation of production of BP-degrading enzyme were prepared as follows. For isolation, Bacto liquefied malt extract agar (Becton, Dickinson Co. Sparks, MD) added with chloramphenicol (50 mg/L) was used. The fungal isolates were maintained on Potato Dextrose Agar medium (PDA, Nihon Pharmaceutical Co., Tokyo, Japan). Fungal minimum medium (FMZ), was used for the selection, evaluation, and production of BP-degrading enzyme. The composition was modified from the Czapek-Dox, a generally used synthetic minimal medium for fungi (0.2% NaNO_3_, 0.1% K_2_HPO_4_, 0.05% MgSO_4_·7H_2_O, 0.05% KCl, 0.001% FeSO_4_·7H_2_O), with 1% emulsified PBSA (Bionolle EM-301) as a sole carbon source instead of sucrose. After autoclaving, filter-sterilized FeSO_4_·7H_2_O was added to prepare FMZ. For selection and evaluation, a two-layered FMZ agarose medium (FMZ-agarose) was prepared in a petri dish (9-cm diameter). The bottom layer was prepared from 10 mL of FMZ (without PBSA) with 1.5% agarose. For the upper layer, 3 mL of 1% emulsified PBSA with 1.5% agarose was poured on to the solidified bottom layer. A liquid FMZ medium was used for the production of enzyme.

### Isolation of phylloplane fungus from gramineous plants

Phylloplane fungi were isolated from healthy leaves of wheat, barley, and rice, which were grown in fields at Tsukuba, Ibaraki, Japan. Leaves were collected 3 times during heading time from 2005 to 2006. On each occasion, 30 flag leaves were cut with sterilized scissors, and were stored individually in sterilized test tubes. Leaf segments (1 × 1 cm) including the margin were cut from the middle part of a blade. The test tubes were then added with 10 mL of sterilized water each, and were shaken at 80 strokes/min for 30 min. Water (1 mL) from each test tube was transferred to petri dishes (9-cm diameter). Liquefied malt extract agar (10 mL; Difco) cooled to 40°C was poured into the petri dish, mixed with the rinse water, and was stand to solidify. Chloramphenicol (50 mg/L) was added to the medium to prevent bacterial growth. After incubation at 25°C for 7 days, colonies of fungi were isolated and stocked on PDA medium.

### Screening of PBSA emulsion-degrading fungi on agarose plate

**Figure 1:**
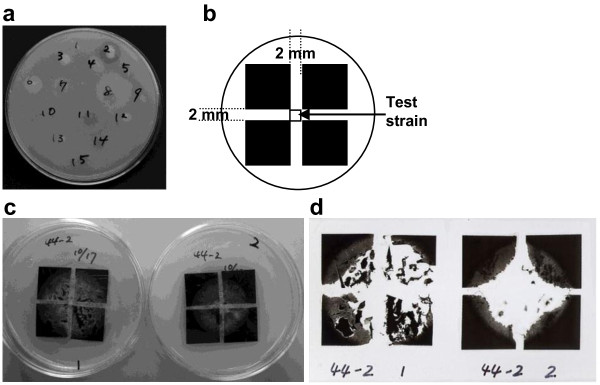
**Screening of BP degrading strains. a**) Typical screening for PBSA emulsion-degrading strains among phylloplane fungi. Typical screening for PBSA and PBS film-degrading fungi on agarose plate: **b**) layout of films and test strain on plate, **c**) images of growth of the strain B44-2 on films during screening for PBSA films (left) and PBS films (right) and **d**) images of degraded films removed from agarose plates.

Microorganisms examined were inoculated on the surface of FMZ-agarose medium and incubated at 28°C for 7 days. A photograph showing a typical screening of a strain for PBSA emulsion-degrading activity is presented in Figure 1a. BP-degrading fungi could decompose emulsified PBSA in the upper layer of the culture plate, resulting in the formation of a clear zone around the fungal colony.

### Screening of PBSA and PBS film-degrading fungi on agarose plate

Evaluation for BP film-degrading activity of filamentous fungi was performed as described in our previous study (Kitamoto et al. [2011]) with a slight modification for filamentous fungi. The typical screening involved the cultivation of the test strain on FMZ-agarose medium at 28°C for 7 days. PBSA or PBS films were cut into 2 × 2 cm squares, sterilized with 70% ethanol, and air-dried in a clean bench. Four pieces of the cut-films were placed on an FMZ-agarose plate side by side forming a bigger square with a distance of 2 mm between two adjacent cut-films (Figure 1b). The FMZ-agarose grown with the test strain was sliced into cubes (2 × 2 × 2 mm) and was inoculated at the center of the 4 cut-films mounted in a dish*.*

Typical screening of a strain was presented in Figure 1c and 1d. After 10 days incubation at 28°C, the cut-films were removed from the agar plate; their images were scanned using a transparency scanner, and their degradation ratios were evaluated by comparing the luminance of the 4 cm^2^ area of the residual film with that of the fresh film as described previously (Kitamoto et al. [2011]).

The areas of the 4 cut-films were determined and averaged, and their degradation rates were evaluated according to the following 5 class-index: I: 0% degradation, no change in film color; II: less than 25% degradation (breakdown of film periphery), loss of film gloss, or thinning of film; III: 26% to 50% degradation; IV: 51% to 90% degradation; and V: over 91% degradation.

### Evaluation of PBSA emulsion-degrading ability of fungi in liquid culture

To screen for production of BP-degrading enzyme, the fungal strains were cultivated in liquid medium as follows. Ten loopfuls of the selected strains were suspended in 20 mL of FMZ liquid medium containing 1% of emulsified PBSA in a glass test tube (21 mm internal diameter) and incubated at 28°C in a reciprocal shaker at 100 rpm for 10 days.

The activity of the PB-degrading enzyme in culture broth was determined as described previously (Kitamoto et al. [2011]) with a slight modification as follows: The supernatant (200 μL ) obtained by filtering the culture broth was added to 1.8 mL of 20 mM Tris–HCl buffer solution (pH 6.8) containing emulsified PBSA in a glass test tube (10 mm internal diameter). The OD_660_ of this mixture was adjusted to approximately 0.5 and then was incubated at 30°C with shaking at 120 rpm. After incubation for 30 min, the percentage transmittance of the BP emulsion was measured with an absorption spectrometer (Spectronic 20A; Shimadzu Co., Tokyo, Japan) at a wavelength of 660 nm against a blank (emulsified PBSA without enzyme). One unit (U) of PBSA emulsion-degrading activity was defined as 1 U/min decrease in absorbance at 660 nm per 1 ml of crude enzyme solution.

### Identification of BP-degrading fungi

The fungal strain, B47-9, was identified by analyzing the genome DNA sequence of internal transcribed spacer (ITS) gene for 18S rRNA as follows: Genomic DNA was extracted using a Genomic DNA Purification Kit (Promega, Fitchburg, WI, USA) according to manufacturer’s instructions. Amplification of internal transcribed spacer ITS - 5.8S rDNA by polymerase chain reaction (PCR) was performed as described by White *et al.* (White et al. [1990]) using the primers ITS1 and ITS4. PCR products were sequenced using an ABI PRISM 3100 DNA sequencer (Applied Biosystems, Foster City, CA, USA).

Sequence data of the ITS region was submitted to the DNA Data Bank of Japan (DDBJ). To identify the isolated strain, the nucleotide sequence of ITS region was compared with those in the DDBJ by using the Blast search with nucleotide sequence database.

### Degradation of BP films on soil by treatment with culture filtrate of strain B47-9

To prepare the culture filtrate, ten loopfuls of the strain B47-9 from a PDA slant were suspended in 100 mL of FMZ liquid medium and incubated in a 300-mL Erlenmeyer flask at 28°C with shaking at 90 rpm for 28 days.

Each test film (PBSA, PBS, and commercially available mulch film) was cut into 10-cm long by 14-cm wide pieces and was placed on 70 g of fertilized soil (Kureha Engeibaido; KUREHA Co.,Tokyo, Japan, pH 6.45), which was either unsterilized or sterilized by autoclaving at 121°C for 15 min in a square petri plate with 10-cm length, 14-cm width, and 1.5-cm depth (AW2000; Eiken Chemical Co., Ltd., Tokyo, Japan). The 28-day old culture broth of strain B47-9 was passed through sterilized gauze to remove the mycelial clumps, and was then poured (70 mL) on to the surface of BP films placed on the soil with a moisture content of 150% of its maximum water holding capacity. After incubation at 28°C for 6 days, the film was removed, washed with distilled water, air-dried and weighed, and was evaluated for the rate of degradation. Experiments were repeated 3 times, and the average weight of the treated films was compared with that of the untreated films.

### Observation of BP film degradation by scanning electron microscopy

To observe the morphology of the fungi present on the surface of the degraded BP film, the film was coated with a gold layer in an ion sputter (Hitachi E-1010, Tokyo, Japan) and was observed under the SEM using JSM-5610LV (JEOL) with an accelerating voltage of 15 kV. The sample was coated with platinum before SEM observation.

### Microbial community analysis of BP film-degrading soil

The effect of treatment of BP film mounted on unsterilized soil with culture filtrate of B47-9 on the transition in the diversity of fungal community was analyzed by comparing the 18S rDNA profiles of soil DNAs created with PCR-DGGE. The analysis was done on soil incubated under 4 different treatment conditions: soil mounted with PBSA film and poured with 70 mL each of either sterilized water or culture filtrate of B47-9; and soil without film treated similarly with either water or the culture filtrate. From each treatment, soil samples (0.4 g) were collected from 3 points: at the center directly under the PBSA film, and at 2 diagonally opposite edges in the square petri plate, at incubation intervals of 0, 7, 14, 21, and 28 days, and immediately stored at – 80°C.

DNA extraction from soil, polymerase chain reaction amplification, and denaturing gradient gel electrophoresis of fungal 18S rDNA fragments were performed as described by Hoshino *et al*. (Hoshino and Morimoto [2008]). The DNA was extracted from 0.4 g of fresh soil using a FastDNA Spin kit for soil (Bio 101, Vista, CA) according to the manufacturer’s recommendations, except that the DNA was eluted in 80 μL DES (DNase/Pyrogen free water; Bio 101) in the final step. Skim milk (80 μL of autoclaved 20% solution) was added to the extraction buffer for soils to avoid DNA adsorption to soil. The DNA was then purified using the Wizard® SV genomic DNA purification system (Promega) according to the manufacturer’s instructions and frozen at −80°C until further analysis. Fungal 18S rRNA genes were amplified from soil DNA using PCR for DGGE using KOD-plus (Toyobo, Osaka, Japan) with primers set NS1 and GCFung according to the conditions described in Bao *et al*. (Bao et al. [2012]). The PCR products were purified and quantified as described in Hoshino *et al*. (Hoshino and Morimoto [2008]), and 100 ng each of the DNA samples were loaded on DGGE based on the method of Muyzer *et al*. (Muyzer et al. [1993]) using the D Code System (Bio-Rad, Hercules, CA). Electrophoresis was performed on a 7% polyacrylamide gel with a denaturing gradient ranging from 20 to 45%, at a running condition set at 50 V at 60°C for 20 h. The molecular marker for fungal DGGE analysis (DGGE Marker IV, Nippon Gene, Toyama, Japan) was used. Gels were stained with SYBR Green I (1:10,000 dilution; FMC BioProducts, Rockland, ME, USA) for 30 min, photographed, scanned and analyzed using the Molecular Imager FX system (Bio-Rad) and stored as TIFF files.

DNA from triplicate soil samples in each treatment condition were analyzed separately. After confirming the reproducibility of the DGGE profiles of the triplicate samples, a representative one was selected. All representative DNA samples were run together on a single a gel for comparison of the differences in their DGGE profiles.

Sequencing of DGGE bands was performed as described in Morimoto *et al.* (Morimoto et al. [2005]), while the PCR amplification for recovering 18 S rRNA gene was done under the original PCR conditions as described above.

## Results

### Isolation of of phylloplane fungi

A total of 1227 strains of phylloplane fungi of various morphologies have been isolated, and among them, 55 strains (4.5%) were selected for having degraded PBSA emulsion. Of the 55 strains, 43 (78.2%), and 37 (67.3%) degraded PBSA and PBS films, respectively, on FMZ agarose plate (Table 1). All the strains which degraded PBS films also degraded PBSA films. Among them, 4 strains degraded over 90% of PBSA film, and hence, were classified in group V, of the 4 strains, only strain B47-9 exhibited high degradation rate for PBSA film (91.2 ± 1.64 %) and PBS film (90.9 ± 1.17%).

**Table 1:** Classification of PBSA and PBS film-degrading fungi on agarose plate based on degradation rates

**Class-index**^**a**^	**I**	**II**	**III**	**IV**	**V**
PBSA	12	19	8	12	4
PBS	18	29	5	2	1

^**a**^Class-index: I: 0% degradation, no change in film color; II: less than 25% degradation (breakdown of film periphery), loss of film gloss, or thinning of film; III: 26% to 50% degradation; IV: 51% to 90% degradation; and V: over 91% degradation.

### Screening for BP-degrading enzyme expression in liquid culture

The 55 PBSA emulsion-degrading strains were also tested for their ability to produce BP- degrading enzyme by cultivating them in liquid FMZ medium. After incubation at 28°C for 10 days, only the culture broth of strain B47-9 showed loss of turbidity, which is indicative of degradation of PBSA emulsion. The PBSA emulsion degradation activity of the supernatant of each culture broth was also analyzed. After 10 days cultivation, the activity (0.19 U /ml) was detected only in the supernatant of strain B47-9.

### Identification of BP-degrading fungi

Strain B47-9 was isolated from barley in May 2006 in Tsukuba, Ibaraki, Japan. This strain has not been morphologically identified because it did not form any spores in spite of inducing its sporulation under various culture conditions. To identify this strain, a comparison of the DNA sequence of its ITS1–5.8S rDNA region (DDBJ accession no. AB693768) with those at DDBJ was done. The results revealed that the sequence concerned of this strain showed 97% homology to that of a strain of *Paraphoma chrysanthemicola* (FJ426987), which belong to Phaeosphaeriaceae (Aveskamp et al. [2009]).

### Degradation of BP mulch films mounted on soil by culture filtrate

To observe the acceleration of degradation of BP mulch film on soil by strain B47-9, sterilized BP films (PBSA, PBS, and commercially available BP mulch film) both on unsterilized or sterilized soil were treated with B47-9 culture filtrate. The culture filtrate was confirmed by dilution plate technique to have a density of 3.6 ± 0.49 CFU/mL and a BPSA emulsion degradation activity of 0.28 U/mL.

**Figure 2:**
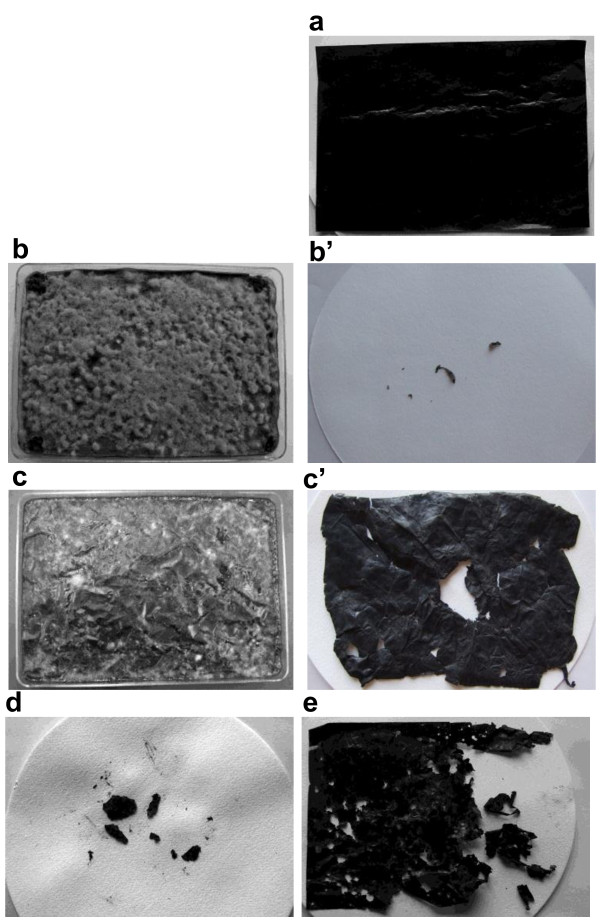
**Degradation of BP films by treatment of strain B47-9 on soil. a**) Control film (PBSA) without treatment, and films 6 days after treated with culture filtrate of the strain B47-9: commercially available BP mulch film **b**) on sterilized soil, and **b**’) recovered film from sterilized soil, and the same film treated **c**) on unsterilized soil, and **c**’) recovered film from unsterilized soil, **d**) PBSA film and **e**) PBS film, recovered from unsterilized soil.

During the incubation of the treated films, fungal mycelia gradually spread and covered the surface of the BP mulch films. After 6 days incubation of commercially available films on sterilized soil, their surfaces became thoroughly covered with fluffy thick gray-colored mycelia (Figure 2b). The films were degraded intensively based on the weight loss of recovered residual film (Figure 2b’) which accounts for 99.8 (SD = 0.11)% of their initial weight. On the other hand, the surfaces of the same films on unsterilized soil were only sparsely covered with gray mycelia interspersed with several fungal colonies of different colors (Figure 2c). The average weight loss of the films was 14.6% (SD = 2.18) (Figure 2c’), indicating that the degradation process was slower on unsterilized soil than on sterilized soil. The weight losses of the PBSA and PBS films in the same treatment on unsterilized soil were 91.2% (SD = 1.64) and 23.7% (SD = 3.10), respectively (Figure 2d, e).

### Observation of BP mulch film degradation by scanning electron microscopy

**Figure 3:**
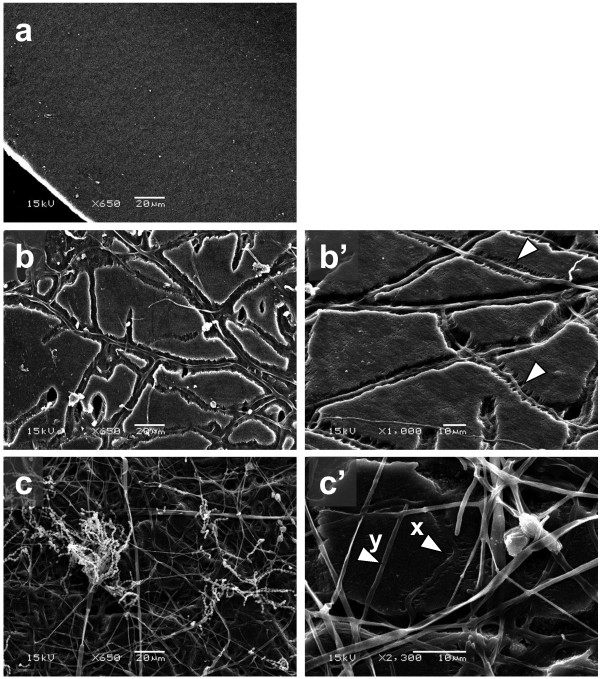
**Scanning electron micrograph for the surface of commercially available BP mulch film 6 days after treatment of strain B47-9 on soil. a**) Control film without treatment (×650), and treated film **b**) on sterilized soil (×650), and **b**’) its magnified photograph (×1000), treated film on **c**) unsterilized soil (×650), and **c**’) its magnified photograph (×2300).

SEM examination of the degraded film on sterilized soil revealed the presence of breaks along the lines where fungal growth was observed (Figure 3b). To examine the degraded part in more detail, magnified SEM photographs of the films were taken (Figure 3b’). On the surface of the film mounted on unsterilized soil, networks of hyphae and presence of breaks (Figure 3c) were also observed similar to those shown in Figure 3b. Furthermore, holes of varying sizes were also seen in the film directly underneath the hyphal networks, which were found to have piled up around bigger holes. We also found conidia which are morphologically identical to those of *Penicillium* spp. In a magnified part of the same film shown in Figure 3c’, film-degrading hyphae (**x**) and non-degrading hyphae (**y**) were observed.

### Microbial community analysis of BP mulch film degrading soil

**Figure 4:**
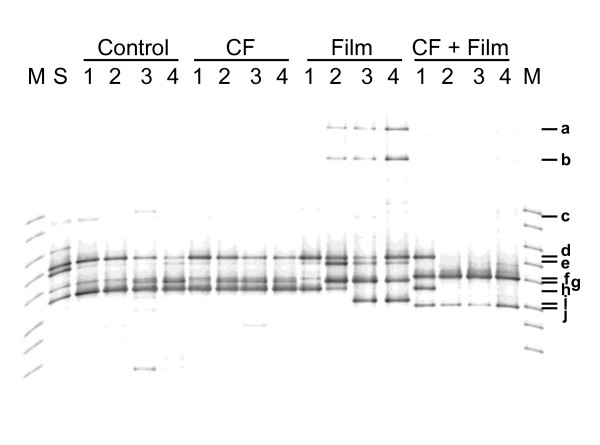
**Transition of microbial diversity in soil during PBSA film degradation.** PCR-DGGE profile of 18S rDNA of soil treated with water (control), culture filtrate, PBSA film and water, and PBSA film and culture filtrate. **M**: marker, **S**: untreated soil. Number 1 to 4 means treatment week. Closely related species based on the similarity in the sequence of the same band are as follows: **a**, *Asperigillus* sp., **b**, *Penicillium* sp., **c**. *Paecilomyces* sp., **d**., *Penicillium decumben*, **f**. strain B47-9, **g**, *Isaria takamizusanensis* or *Paecilomyces lilacinu*, **h**., *Paecilomyces* sp. , **e**.**i**. and **j**, *Acanthamoeba* sp.

To further confirm the contribution of inoculated strain B47-9 to the film degradation on unsterilized soil, the transition in the fungal diversity of unsterilized soil was analyzed by PCR-DGGE. DGGE profiles under all test conditions are displayed in Figure 4. Bands with the same mobility and sequences are considered to have been derived from the same strain. The sequence data for the ten major bands (**a** to **j**) were registered in DDBJ (accession nos. AB727580 - AB727589, respectively). Species with closely related sequences for the same band were identified from DDBJ, and were listed in the figure legend.

In treatments without PBSA film, no remarkable differences in the number and profiles of bands between samples treated with water and culture filtrate were observed. Bands (**d, g and h**) were stably detected in all soil samples without film. They have been identified as closely related to *Penicillium decumbens* (d), a strain of *Isaria takamizusanensis* strain or *Paecilomyces lilacinu* (g), and *Paecilimyces* sp. strain (h).

Differences in the DGGE profiles were observed in the soil samples covered with PBSA film. The **d** and **g** bands were stably continuously during the whole incubation period, but not band **h,** which disappeared after 3 and 4 weeks incubation. Three new bands (**a, b** and **e**) appeared after 2 weeks incubation, and they were found to be closely related to *Aspergillus* sp., *Penicillium* sp., and *Acanthamoeba* sp. strains, respectively. They have been continuously detected in the samples incubated for 2 to 4 weeks. Another new band (**i**), related to *Acanthamoeba* sp. strain, was also detected in the 3 and 4 week-incubated samples.

In contrast, the DGGE profile of PBSA film-covered soil sample treated with B47-9 culture filtrate was quite different from those of other samples. The band (**g**) for soil-derived microorganisms was not detected after 1 week incubation. In addition, new bands (**f**) and (**j**) for B47-9 and *Acanthamoeba* sp., respectively, were observed. The bands (**d**) and (**h**) for soil-derived *Penicillium decumben* and *Paecilomyces* sp., respectively, disappeared after 2 weeks incubation, but those of B47-9 (**f**) and *Acanthamoeba* (**j**) remained detectable and in fact appeared as dominant bands.

## Discussion

In the present study, we determined the potential of using BP degrading- microorganisms for accelerating the degradation of used BP mulch films in agricultural fields. Several studies concerning fungi that possess the ability to degrade plastics have already been reported. A *Fusarium solani* f. sp. *pisi*, a plant pathogenic fungus degraded BP of poly (ϵ-caprolactone) (PCL) emulsion (Murphy et al. [1996]). Heterologously expressed cutinases of plant pathogenic fungi (*F. solani* f. sp. *pisi, Alternaria brassicicolaAspergillus fumigates* and *Humicola insolens*) degraded PCL film (Baker et al. [2012]). *Aspergillus oryzae*, a fungus widely used in traditional Japanese fermentation industries, degraded PBS and PBSA mulch film (Maeda et al. [2005]). To address safety concerns regarding the use of microorganisms in the agricultural fields, in this study, we performed our microbial isolation from gramineous crop plants. Our results showed that 4.5% of the isolated fungal strains degraded PBSA emulsion. We have previously reported in a related study that 2% to 100% of yeast populations isolated from seed rice husks degraded PBSA emulsion (Kitamoto et al. [2011]). These findings thus demonstrate that phylloplane is an important source of BP-degrading eukaryotic microorganisms. Further analysis and identification of the evaluated fungal strains could be expected to give us detailed information about the diversity and activities of BP-degrading phylloplane-derived fungi.

Among the isolated strains, B47-9 was found to have the strongest ability to degrade PBSA and PBS films on agarose medium, and was the only strain that resulted to a detectable PBSA emulsion-degrading activity in liquid culture. Strain B47-9 is identified as an imperfect fungi belonging to Ascomycota, and is closely related to *Phoma*. To the best of our knowledge, this is the first report on a *Phoma*-related fungus which possesses a BP-degrading ability.

Strain B47-9 degraded almost all the commercially available polymer-blended BP mulch film mounted on indoor-sterilized soil environment (Figure 2b’). Our analysis of the commercially available mulch film used in this study revealed that it is composed of PBS, PBSA, and PBAT at a weight-based ratio of 47:37:17. This observation showed that strain B47-9 can degrade various BP components. On the unsterilized soil, the lower degradability of the polymer-blended commercially available mulch film (Figure 2c’) compared with PBSA (Figure 2d) and PBS (Figure 2e) films indicated that PBAT is degraded more slowly than PBSA and PBS in natural soil environment.

We observed how the strain grew on the film surface and degraded the film on sterilized soil. Vigorous growth of gray-colored mycelia of B47-9 was observed on the surface of polymer blended film (Figure 2b). Under the SEM, we observed the strain grew on the surface of film, producing breaks and holes on the film along the direction of its hyphal growth (Figure 3b, b’). These observations support our assumption that B47-9 may have secreted an enzyme capable of directly degrading the film. As indicated by the arrows in figure 3b’, it was clear that the degradation event proceeded along one direction of the film. The same degradation characteristics was also observed under SEM in poly [(*R*)-3-hydroxyputyrate] (p(3HB)) film after partial degradation by PHB depolymerase (Iwata [2005]). It is well known that the amorphous region is etched faster than the crystal one (Vert [2005]). BP-degrading enzyme of strain B47-9 may have also degraded the polymer blended mulch film in a similar way.

On the surface of the film mounted on unsterilized soil, networks of mycelia and conidia of soil-derived *Penicillium* sp. were observed (Figure 3c). A magnified part of the same film is shown in Figure 3c’, where the arrow (**x**) indicates the film-degrading hyphae, which we presumed to be strain B47-9, along with other hyphae that did not degrade the film, designated by arrow (**y**), which were believed to be soil-derived microorganisms. These observations support our presumption regarding the existence of microbial competition between strain B47-9 and native soil fungi.

To further confirm the contribution of inoculated strain B47-9 to the film degradation on unsterilized soil, the transition in the fungal diversity of unsterilized soil as well as the growth of B47-9 were analyzed by PCR-DGGE. The populations of these filamentous fungal species (*Aspergillus* sp., *Penicillium* sp.) and *Acanthamoeba* sp., which commonly inhabit the soil (Rosenberg et al. [2009]), were found to increase in the presence of PBSA film.

In contrast, in the PBSA film-covered soil sample treated with B47-9 culture filtrate, the following bands were detected after 1 week incubation: **d** and **h** (soil-delived fungi), **f** (B47-9) and **j** (*Acanthamoeba*). These results corresponded to our SEM observations of the degraded film surface as described earlier. The bands for soil-derived fungi disappeared after 2 weeks incubation, and this was construed as due to the degradative activity of native soil fungi. Those of B47-9 (**f**) and *Acanthamoeba* (**j**), however, were still detected, which in fact, appeared as dominant bands. These results indicated that strain B47-9 could remain dominant in the soil only when PBSA film was mounted on it. Furthermore, increments of soil-derived *Acanthamoeba* sp. strains were observed only when the soil was covered with PBSA film. *Acanthamoeba* is found in a variety of soil, and has been reported to assist in rapidly changing the composition of bacterial community in the soil (Rosenberg et al. [2009]) by increasing the digestible nutrients which support the growth of soil microorganisms, and transporting and dispersing microorganisms into new environmental niches (Schuster [2002]). Furthermore, *Acanthamoeba* spp. strains possess degradative activities for soil microorganisms, such as yeasts and fungi (Steenbergen et al. [2001]). There is also a report regarding a strain of *Acanthamoeba castellani* which is known to have depolymerizing activity for polyhydrozybutyarate (PHB) (Anderson et al. [2005]), a bacterial storage polyester. It is, therefore, possible that *Acanthamoeba* spp. strains observed in this study prefer to grow around PBSA film, and any of them may have contributed to the changes in microbial community composition.

In this study, we isolated and confirmed that a fungal strain B47-9 has directly contributed to accelerating the degradation of soil-mounted film. Since this strain B47-9 was isolated from healthy leaf of barley, it may be assumed that it can be safely utilized for acceleration of degradation of BP mulch film after use. The establishment of an economical and environment-friendly technique for BP degradation using fungi, such as strain B47-9, could be expected to provide a viable solution to the plastic disposal problem in agriculture in the future.

## Competing interests

The authors declare that they have no competing interests.

## References

[B1] Abe M Kobayashi K Honma N Nakasaki K Microbial degradation of poly(butylene succinate) by Fusarium solani in soil environments Polym Degrad Stab 2010 95 2 138 143 DOI 10.1016/j.polymdegradstab.2009.11.042

[B2] Anderson IC Cairney JW Diversity and ecology of soil fungal communities: increased understanding through the application of molecular techniques Environ Microbiol 2004 6 8 769 779 10.1111/j.1462-2920.2004.00675.x 15250879

[B3] Anderson IJ Watkins RF Samuelson J Spencer DF Majoros WH Gray MW Loftus BJ Gene discovery in the Acanthamoeba castellanii genome Protist 2005 156 2 203 214 10.1016/j.protis.2005.04.001 16171187

[B4] Aveskamp MM Verkley GJM de Gruyter J Murace MA Perello A Woudenberg JHC Groenewald JZ Crous PW DNA phylogeny reveals polyphyly of Phoma section Peyronellaea and multiple taxonomic novelties Mycologia 2009 101 3 363 382 10.3852/08-199 19537209

[B5] Baker PJ Poultney C Liu Z Gross R Montclare JK Identification and comparison of cutinases for synthetic polyester degradation Appl Microbiol Biotechnol 2012 93 1 229 240 10.1007/s00253-011-3402-4 21713515

[B6] Bao Z Ikunaga Y Matsushita Y Morimoto S Takada-Hoshino Y Okada H Oba H Takemoto S Niwa S Ohigashi K Suzuki C Nagaoka K Takenaka M Urashima Y Sekiguchi H Kushida A Toyota K Saito M Tsushima S Combined analyses of bacterial, fungal and nematode communities in andosolic agricultural soils in Japan Microbes Environ 2012 27 1 72 79 10.1264/jsme2.ME11281 22223474 PMC4036027

[B7] Gross RA Kalra B Biodegradable polymers for the environment Science 2002 297 5582 803 807 10.1126/science.297.5582.803 12161646

[B8] Heredia A Biophysical and biochemical characteristics of cutin, a plant barrier biopolymer Bba-Gen Subjects 2003 1620 1–3 1 7 10.1016/S0304-4165(02)00510-X 12595066

[B9] Hoshino YT Matsumoto N An improved DNA extraction methods using skimmed milk from soils that strongly adsorb DNA Microbes Environ 2004 19 13 19 10.1264/jsme2.19.13

[B10] Hoshino YT Morimoto S Comparison of 18S rDNA primers for estimating fungal diversity in agricultural soils using polymerase chain reaction-denaturing gradient gel electrophoresis Soil Sci Plant Nutr 2008 54 5 701 710 10.1111/j.1747-0765.2008.00289.x

[B11] Iwata T Strong fibers and films of microbial polyesters Macromol Biosci 2005 5 8 689 701 10.1002/mabi.200500066 16052600

[B12] Kasuya K Ishii N Inoue Y Yazawa K Tagaya T Yotsumoto T Kazahaya J Nagai D Characterization of a mesophilic aliphatic-aromatic copolyester-degrading fungus Polym Degrad Stab 2009 94 8 1190 1196 10.1016/j.polymdegradstab.2009.04.013

[B13] Kitamoto HK Shinozaki Y Cao XH Morita T Konishi M Tago K Kajiwara H Koitabashi M Yoshida S Watanabe T Sameshima-Yamashita Y Nakajima-Kambe T Tsushima S Phyllosphere yeasts rapidly break down biodegradable plastics AMB Express 2011 1 1 44 10.1186/2191-0855-1-44 22126328 PMC3293741

[B14] Kyrikou I Briassoulis D Biodegradation of agricultural plastic films: A critical review J Polym Environ 2007 15 3 227 10.1007/s10924-007-0063-6

[B15] Maeda H Yamagata Y Abe K Hasegawa F Machida M Ishioka R Gomi K Nakajima T Purification and characterization of a biodegradable plastic-degrading enzyme from Aspergillus oryzae Appl Microbiol Biotechnol 2005 67 6 778 788 10.1007/s00253-004-1853-6 15968570

[B16] Morimoto S Togami K Ogawa N Hasebe A Fujii T Analysis of a bacterial community in 3-chlorobenzoate-contaminated soil by PCR-DGGE targeting the 16S rRNA gene and benzoate 1,2-dioxygenase gene (benA) Microbes Environ 2005 3 151 159

[B17] Murphy CA Cameron JA Huang SJ Vinopal RT Fusarium polycaprolactone depolymerase is cutinase Appl Environ Microbiol 1996 62 2 456 460 8593048 10.1128/aem.62.2.456-460.1996 PMC167813

[B18] Muyzer G de Waal EC Uitterlinden AG Profiling of complex microbial populations by denaturing gradient gel electrophoresis analysis of polymerase chain reaction-amplified genes coding for 16S rRNA Appl Environ Microbiol 1993 59 3 695 700 7683183 10.1128/aem.59.3.695-700.1993 PMC202176

[B19] Park D Phylloplane fungi - Tolerance of hyphal tips to drying T Brit Mycol Soc 1982 79 174 178 10.1016/S0007-1536(82)80212-X

[B20] Rosenberg K Bertaux J Krome K Hartmann A Scheu S Bonkowski M Soil amoebae rapidly change bacterial community composition in the rhizosphere of Arabidopsis thaliana ISME J 2009 3 6 675 684 10.1038/ismej.2009.11 19242534

[B21] Sang BI Hori K Tanji Y Unno H Fungal contribution to in situ biodegradation of poly(3-hydroxybutyrate-co-3-hydroxyvalerate) film in soil Appl Microbiol Biotechnol 2002 58 2 241 247 10.1007/s00253-001-0884-5 11876418

[B22] Sangwan P Wu DY New insights into polylactide biodegradation from molecular ecological techniques Macromol Biosci 2008 8 4 304 315 10.1002/mabi.200700317 18383571

[B23] Sangwan P Way C Wu DY New insight into biodegradation of polylactide (PLA)/clay nanocomposites using molecular ecological techniques Macromol Biosci 2009 9 7 677 686 10.1002/mabi.200800276 19148900

[B24] Schuster FL Cultivation of pathogenic and opportunistic free-living amebas Clin Microbiol Rev 2002 15 3 342 354 10.1128/CMR.15.3.342-354.2002 12097243 PMC118083

[B25] Steenbergen JN Shuman HA Casadevall A Cryptococcus neoformans interactions with amoebae suggest an explanation for its virulence and intracellular pathogenic strategy in macrophages Proc Natl Acad Sci U S A 2001 98 26 15245 15250 10.1073/pnas.261418798 11742090 PMC65014

[B26] Tan FT Cooper DG Maric M Nicell JA Biodegradation of a synthetic co-polyester by aerobic mesophilic microorganisms Polym Degrad Stab 2008 93 8 1479 1485 10.1016/j.polymdegradstab.2008.05.005

[B27] Vert M Aliphatic polyesters: great degradable polymers that cannot do everything Biomacromolecules 2005 6 2 538 546 10.1021/bm0494702 15762610

[B28] White TJBT Lee SB Taylor JW Innis MA, Gelfand DH, Sninsky JJ, White TJ Amplification and direct sequencing of fungal ribosomal RNA genes for phylogenetics PCR protocols, a guide to methods and applications 1990 Academic Press, San Diego 315 322

[B29] Xu J Guo BH Poly(butylene succinate) and its copolymers: research, development and industrialization Biotechnol J 2010 5 11 1149 1163 10.1002/biot.201000136 21058317

